# Problematic Social Media Use and the Digital Dependency–Distress Cascade: An Urgent Call for Academic and Public Health Action

**DOI:** 10.1002/brb3.71024

**Published:** 2025-10-31

**Authors:** MD. Faisal Ahmed

**Affiliations:** ^1^ Department of Health Science and Informatics Bangladesh Institute of Innovative Health Research Mirpur, Dhaka Bangladesh

**Keywords:** anxiety disorders, nomophobia, screen time, sleep disorders

To the Editor,

The recent article by Islam et al. (2025) on Problematic Social Media Use (PSMU) Among University Entrance Test‐Takers deserves urgent and serious attention—not merely as a study of digital behavior, but as a sentinel warning of a public mental health emergency unfolding in real time. The authors’ finding that one in five admission test‐takers in Bangladesh meet criteria for PSMU, with stress exerting both direct and indirect effects through prolonged online exposure, reframes excessive digital engagement as a psychological risk architecture rather than a passive lifestyle choice.

What makes these findings deeply concerning is their convergence with a growing body of evidence across low‐ and middle‐income countries. Das et al. (2025) report that screen exposure exceeding 4 h daily sharply elevates anxiety and depression, while Md. F. Ahmed, Shahariar et al. (2025) reveal that nearly half of Bangladeshi university students experience severe nomophobia, with profound effects on mental well‐being and insomnia. Rahman et al. (2025) further demonstrate that social media addiction, fatigue, and fear of missing out (FoMO) act as behavioral accelerants, degrading sleep quality and emotional stability, whereas O. Ahmed, Dawel et al. (2025) identify insomnia as the critical mediator linking PSMU to anxiety and depression. Importantly, Tung et al. (2025) show that nomophobia not only amplifies psychological distress but also operates as a transnational phenomenon, with gendered vulnerabilities evident across Asia and Europe. Taken together, these findings delineate what we term the Digital Dependency–Distress Cascade: a self‐reinforcing cycle in which hyperconnectivity, academic pressure, and algorithmically engineered engagement coalesce to erode mental health, cognitive resilience, and sleep integrity (Md. F. Ahmed 2025). Alarmingly, this cascade now transcends geography, gender, and socioeconomic status—yet institutional responses remain piecemeal, failing to match the accelerating scale of risk.

What is needed is not incremental awareness, but a paradigm shift:

- Mandatory digital mental health curricula to cultivate critical digital citizenship;
- Systematic screening for nomophobia, PSMU, and sleep disorders within academic health systems;
- Digital detox interventions addressing algorithmic overexposure and academic burnout; and
- Policy frameworks recognizing hyperconnectivity as a determinant of youth mental health on par with substance use or sedentary behavior.

Islam et al. (2025) have provided a crucial empirical foundation. It is now imperative to pursue longitudinal, mechanistic, and intervention‐focused research capable of disrupting this cascade before it hardens into a defining mental health crisis of the digital era.

**MD. Faisal Ahmed**

## Author Contributions

**MD. Faisal Ahmed**: conceptualization, writing – original draft, writing – review & editing.

## Funding

The author has nothing to report.

## Conflicts of Interest

The author declares no conflicts of interest.

## Peer Review

The peer review history for this article is available at https://publons.com/publon/10.1002/brb3.71024

## Data Availability

No new data were generated or analyzed in this study. All supporting materials and references are publicly available as cited in the article.

## References

[brb371024-bib-0001] Ahmed, Md. F. 2025. “Rethinking Digital Mental Health: From Nomophobia to Neurocognitive Exhaustion in LMIC Youth.” Health Science Reports 8, no. 9: e71278. 10.1002/hsr2.71278. 40988897 PMC12451051

[brb371024-bib-0002] Ahmed, Md. F. , M. Shahariar , F. I. Shila , J. R. Banik , and F. Afifa . 2025. “Assessing the Relationships Between Nomophobia, Psychological Well‐Being, and Insomnia in Bangladeshi Youth.” Journal of Public Health 1–10. 10.1007/s10389-025-02508-y.39932724

[brb371024-bib-0003] Ahmed, O. , A. Dawel , E. I. Walsh , and N. Cherbuin . 2025. “Longitudinal Associations Between Problematic Social Media Use and Mental Health: Mediating Role of Sleep.” Addictive Behaviors 170: 108446. 10.1016/j.addbeh.2025.108446. 40782602

[brb371024-bib-0004] Das, S. , A. A. Zubayer , M. F. Snigdha , et al. 2025. “Social Media Use Time and Mental Health of Young Adults: A Cross‐Sectional Study in Bangladesh.” Health Science Reports 8, no. 7: e71117. 10.1002/hsr2.71117. 40709074 PMC12286897

[brb371024-bib-0005] Islam, M. M. , S. M. R. Hasan , A. Masuyama , et al. 2025. “Problematic Social Media Use Among University Entrance Test‐Takers: Prevalence, Psychosocial Factors, and a Mediation‐Moderation Model.” Brain and Behavior 15, no. 9: e70911. 10.1002/brb3.70911. 40999592 PMC12463700

[brb371024-bib-0006] Rahman, M. , Md. F. Rabby , Md. R. Kabir , et al. 2025. “Associations Between Social Media Addiction, Social Media Fatigue, Fear of Missing Out, and Sleep Quality Among University Students in Bangladesh: a Cross‐Sectional Study.” Journal of Health, Population and Nutrition 44, no. 1: 152. 10.1186/s41043-025-00896-1. 40349076 PMC12065153

[brb371024-bib-0007] Tung, S. E. H. , W. Y. Gan , W. C. Poon , et al. 2025. “The Mediating Effect of Nomophobia in the Relationship Between Problematic Social Media Use/Problematic Smartphone Use and Psychological Distress Among University Students.” Psychology, Health & Medicine 30, no. 7: 1409–1425. 10.1080/13548506.2025.2478516. 40294608

